# Efficacy and safety of stem cell therapy in patients with dilated cardiomyopathy: an umbrella review of systematic reviews

**DOI:** 10.1097/JS9.0000000000001142

**Published:** 2024-02-06

**Authors:** Jun Ran, Arkadiusz Dziedzic, Israa Habeeb Naser, Ramaiah Itumalla, Jeetendra Kumar Gupta, Sarvesh Rustagi, Prakasini Satapathy, Mahalaqua Nazli Khatib, Shilpa Gaidhane, Quazi Syed Zahiruddin, Abhay M Gaidhane, Ranjit Sah

**Affiliations:** a Department of Cardiovascular Surgery, Fuwai Hospital, National Center for Cardiovascular Diseases, Chinese Academy of Medical Sciences and Peking Union Medical College, Beijing, China; b Department of Conservative Dentistry with Endodontics, Medical University of Silesia, Katowice, Poland; c Medical Laboratories Techniques Department, AL-Mustaqbal University, Hillah, Babil, Iraq; d School of Management, The Apollo University, Chittoor, Andhra Pradesh; e Institute of Pharmaceutical Research, GLA University Mathura, Uttar Pradesh; f School of Applied and Life Sciences, Uttaranchal University; g School of Pharmacy, Graphic Era Hill University, Dehradun, India; h Center for Global Health Research, Saveetha Medical College and Hospital, Saveetha Institute of Medical and Technical Sciences, Saveetha University, Chennai; i Division of Evidence Synthesis; j South Asia Infant Feeding Research Network (SAIFRN), Division of Evidence Synthesis, Global Consortium of Public Health and Research; k One Health Centre (COHERD), Jawaharlal Nehru Medical College; l Jawaharlal Nehru Medical College, and Global Health Academy, School of Epidemiology and Public Health. Datta Meghe Institute of Higher Education, Wardha; m Department of Clinical Microbiology, DY Patil Medical College, Hospital and Research Centre, DY Patil Vidyapeeth, Pune, Maharashtra, India; n Tribhuvan University Teaching Hospital, Kathmandu 46000, Nepal

**Keywords:** Stem cell therapy, Dilated cardiomyopathy, umbrella review, meta-analysis

## Abstract

**Background:**

Stem cell therapy (SCT) has emerged as a potential therapeutic avenue, with various cell types being explored for their efficacy in treating dilated cardiomyopathy (DCM). However, the safety and efficacy of these therapies have been the subject of numerous systematic reviews. This umbrella review aims to consolidate the existing evidence on stem cell interventions for DCM, providing a comprehensive overview of the current research landscape.

**Methods:**

This review was conducted following the JBI and PRISMA guidelines. Systematic reviews and meta-analyses of randomized controlled trials (RCTs) evaluating the safety and efficacy of SCT for DCM were included. Outcomes such as 6-minute walk test (6-MWT), left ventricular end-diastolic diameter (LVEDD), left ventricular ejection fraction (LVEF), major adverse cardiovascular events (MACE), New York Heart Association (NYHA), and quality of life (QoL), among others, were considered. A literature search was executed across databases like PubMed, Embase, Web of Science, and Cochrane Database up to 7 October 2023. The quality of the included reviews was assessed using the JBI Checklist for Systematic Reviews and Research Syntheses. Data synthesis was carried out in both narrative and tabular formats, with the GRADE criteria guiding the determination of evidence certainty.

**Results:**

Nine systematic reviews met the inclusion criteria. LVEF found to be significantly improved with SCT. LVEDD and LVEDV assessments yielded mixed results, with some reviews observing significant changes. Left ventricular end-systolic volume showed consistent reductions across multiple studies. B-type natriuretic peptide concentrations post-interventions were explored in several studies, with mixed findings. Health-related quality of life (HRQL) showed varied results, with some studies noting improvements and others finding no significant differences. NYHA classifications and 6-MWT results indicated potential benefits from stem cell treatments. SCT was observed to be generally safe. The certainty of evidence was low or very low for most of outcomes.

**Conclusion:**

SCT showed has shown promise in treating DCM, with many studies highlighting its safety and potential benefits. Nonetheless, the existing data has its limitations due to biases in the RCTs studies. To truly establish the benefits of SCT for DCM, future high-quality RCTS, are crucial.

## Introduction

Highlights

- Stem cell therapy (SCT) for dilated cardiomyopathy shows significant improvement in left ventricular ejection fraction.
- Evidence on the impact of SCT on heart structure measures such as left ventricular end-diastolic diameter and left ventricular end-systolic volume is mixed.
- Patient functionality may improve with SCT, as suggested by New York Heart Association classifications and 6-minute walk test scores.
- Safety profile of SCT is favourable, yet high-quality randomized controlled trials are essential to validate these findings due to the current low certainty of evidence.

Dilated cardiomyopathy (DCM) is a significant heart muscle disorder typified by dilation and impaired systolic function of the left or both ventricles. This condition arises independently of coronary artery disease, hypertension, or valvular disorders^1,2^. Despite its global prevalence and being a leading reason for heart transplants among adults^3–6^, DCM’s treatment remains challenging. While optimal medical interventions have evolved since the early 1990s, offering some respite, the overall deterioration of left ventricular function persists in many patients^5,7^.

Stem cell therapy (SCT) presents a promising frontier in DCM treatment. Stem cells possess unique attributes around 2001^8^. like self-renewal, proliferation, regeneration, and potential for varied lineage differentiation^9^. The overarching goal with SCT for DCM is either to rejuvenate the cardiac muscle or activate natural repair mechanisms, primarily by replacing the deceased myocardium or stimulating physiological mechanisms of repair^10^. The journey of cell-based treatments for heart ailments began with the transplantation of skeletal myoblasts in the human myocardium. Numerous trials have since experimented with cell-based solutions for both ischaemic and non-ischaemic heart failures^10–15^. A myriad of stem cell types, both autologous and allogeneic, have been explored for treating heart failure due to ischaemic cardiomyopathy and DCM. This includes haematopoietic stem cells, skeletal myoblasts, cardiac stem cells mesenchymal stem cells, and cardiosphere-derived cells^10^. Collection methods for these stem cells vary. Haematopoietic stem cells, for instance, are gathered from venous blood post a mobilization regimen, usually leveraging granulocyte colony-stimulating factor (G-CSF). In contrast, bone marrow-derived stem cells can be directly procured from a bone marrow aspirate, typically taken from the patient’s ilium^16^. Subsequent administration of these cells can happen through various delivery channels, including coronary arteries, coronary sinus, peripheral veins, or direct intramyocardial injections using surgical or trans endocardial methods^17–22^.

However, the efficacy and safety of these varied stem cell therapies remain a topic of numerous systematic reviews^23–26^. Umbrella review synthesizes evidence from multiple systematic reviews on a topic to provide a comprehensive understanding of the available research. Its primary purpose is to offer a broader and clearer perspective on a particular subject by collating and analyzing findings from various systematic reviews, ensuring that decision-makers have a consolidated and high-level overview of the evidence^27,28^. The purpose of this umbrella review is to consolidate the existing evidence related to stem cell interventions for DCM, offering a comprehensive overview of the current landscape. This summation is essential to understand the nuances of different treatments and identify the most promising directions for future research and clinical application.

## Methods

This umbrella review was conducted in accordance with the JBI (Joanna Briggs Institute)^29^ and PRISMA, Supplemental Digital Content 1, http://links.lww.com/JS9/B817 (Preferred Reporting Items for Systematic Reviews and Meta-Analyses) guidelines (Table S1, Supplemental Digital Content 2, http://links.lww.com/JS9/B818)^30,31^. The review has been registered with PROSPERO. We used AMSTAR-2, Supplemental Digital Content 3, http://links.lww.com/JS9/B819 to evaluate the quality of the present review, and it scored high quality^32^ (Table S2, Supplemental Digital Content 2, http://links.lww.com/JS9/B818). The study also registered with Research Registry (UIN: reviewregistry1781).

### Selection criteria

This umbrella review incorporates systematic reviews and meta-analyses of randomized controlled trials (RCTs) that assessed the safety and efficacy of SCT for dilated cardiomyopathy. We didn’t put any restriction on the range of outcome for safety and efficacy. outcome like 6-minute walk test (6-MWT), left ventricular ejection fraction (LVEF), left ventricular end-diastolic volume (LVEDV), left ventricular stroke volume (LVSV), major adverse cardiovascular events (MACE), left ventricular end-diastolic diameter (LVEDD), New York Heart Association (NYHA), quality of life (QoL) are assessed in previous reviews. The following were excluded from this review: observational studies, case reports, case series, animal studies, conference abstracts, and narrative reviews. Articles not available in English were also excluded. Refer Table S3, Supplemental Digital Content 2, http://links.lww.com/JS9/B818 for detailed inclusion criteria.

### Literature search and screening

An electronic literature search was conducted across the following databases: PubMed, Web of Science, Embase, and the Cochrane Database, to identify systematic reviews on the topic up to 7 October 2023. Keywords and MeSH terms related to “stem cells”, “dilated cardiomyopathy”, “systematic review”, and “meta-analysis” were used in the search. There were no restrictions placed on the year of publication. Table S4, Supplemental Digital Content 2, http://links.lww.com/JS9/B818 provides search strategy.

Two independent reviewers screened the results retrieved from the databases after duplicates were removed using the Nested Knowledge software. An initial screening was conducted based on titles and abstracts, followed by a full-text review. Any differences of opinion regarding the inclusion of articles were resolved by consulting a third reviewer.

### Data extraction

Data extraction was performed by two reviewers. They first extracted data from each eligible systematic review. Information such as author name, year of publication, databases and search year, number and type of included studies, year of these studies, risk of bias tools used and their results, outcomes of concern, key findings, effect size and CI, *p* value, publication bias, and type of intervention were obtained.

### Quality appraisal

For assessing the quality of the included systematic reviews included in this study, JBI Checklist for Systematic Reviews and Research Syntheses was used^33,34^. The JBI tool offers a comprehensive approach to appraise the methodological quality of systematic reviews. It evaluates various aspects, including the clarity of the research question, the appropriateness of inclusion criteria, and the comprehensiveness of the search strategy, among others.

### Data synthesis

Narrative and tabular formats were used to present the results. If the same outcome was present in different reviews, the review with the largest number of studies, that was most recent, and of the highest methodological quality, was considered. Each outcome was synthesized and summarized separately. Effect estimates, respective CI, and *p* values were presented, accompanied by results on heterogeneity. To determine the certainty of evidence, we followed the Grading of Recommendations Assessment, Development and Evaluation (GRADE) criteria. A summary of evidence table was created, encompassing each outcome. The evidence was classified into four levels of certainty: high, moderate, low, and very low. This classification was based on several factors, including the risk of bias in studies, inconsistency of results, indirectness of evidence, imprecision, and potential publication bias. We utilized the GRADE pro software to compile the summary of evidence table^35^.

## Results

### Literature search

**Figure 1:**
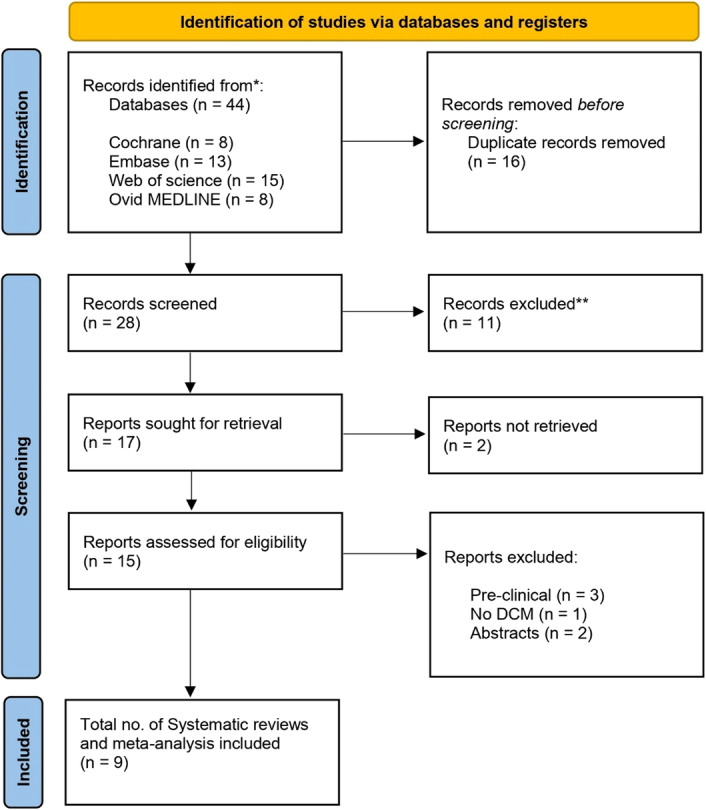
PRISMA flow diagram showing the screening and selection process. DCM, dilated cardiomyopathy.

In the literature search, we identified a total of 44 records from various databases. Before further screening, 16 duplicate records were removed, leaving us with 28 unique records for screening. Of these, 11 records were excluded, reducing the number to 17 potentially relevant reports. These 17 underwent full-text screening, and 9 reviews ultimately met our criteria. The PRISMA flowchart is provided in Fig. 1, showcasing the screening and selection process.

### Characteristics of included reviews

Table 1 summaries the main characteristics of included systematic reviews. The number of included RCTs in these reviews varied significantly, with some reviews including as few as four RCTs and others including as many as 20 RCTs, spanning years from 2006 to 2018^36,38^. All the studies primarily focused on non-ischaemic cardiomyopathy. However, there were variations in the specifics of the disease investigated, with some delving into non-ischaemic DCM and others examining non-ischaemic DCM^25,26^. The interventions largely centred on stem cell therapies derived from bone marrow. In terms of outcomes, there was a wide spectrum across the reviews. Common outcomes like LVEF were evaluated by many reviews^25,40^. Functional outcomes like the six-minute walking test were emphasized by others, and some even considered the Kansas City Cardiomyopathy Questionnaire (KCCQ), a QoL measure^24^. The key findings showed a shared theme of stem cell therapy’s potential to enhance ventricular function. LVEF improvements were noted by several reviews^25,37^. Exercise capacity enhancements and reductions in chamber sizes were highlighted by others^37,40^. However, the impact on mortality showed mixed findings; while some suggested potential mortality rate reductions, others found no significant effect^26,37^. The risk of bias assessment varied across reviews. Some reported a low risk, while others ranged from moderate to high^36,40^. The majority of the reviews did not find significant publication bias.

**Table 1:** Main characteristics of the included reviews.

References	Databases and search	No. included RCTs	Year range of included studies	Type of disease	Type of intervention	Outcomes	Risk of bias of included RCTs	Key findings	Publication bias
Diaz-Navarro *et al*.^23^	Cochrane Library, MEDLINE, and Embase, November 2020	13	2006–2018	NIDCM	BMC	LVEF, Mortality, LVEDV, LVESV, NYHA functional class, QoL using the KCCQ	Overall high risk of bias	BMC transplantation led to significant improvements in LVEF, LVESV, 6-min walk distance, NYHA class, MIHFQ scores, and overall mortality.	No publication bias detected
Lu *et al*.^26^	PubMed, Scopus, and Cochrane CENTRAL, February 20, 2016	7	2010–2015	NIDCM	Bone marrow-derived stem cells	Mortality, LVEF, LVESV, LVEDD, and 6-MWT	Overall risk of bias was moderate to low	Bone marrow stem cell therapy may reduce mortality in patients with NIDCM. However, only a mild LVEF increase was seen during mid-term follow-up, with no boost in exercise capacity.	No publication bias detected
Marquis -Gravel *et al*.^36^	Medline, EBM Reviews, Cochrane CENTRAL, Embase, and ClinicalTrials.gov, 10 November 2013	4	2008–2013	NIDCM	Autologous BMC	LVEF	Low risk of bias	SC therapy might improve LVEF, but not LVEDD, for the treatment of non-ischaemic CMP	No publication bias detected
Nso *et al*.^25^	PubMed, Scopus, and Cochrane CENTRAL	11	2010–2017	NIDCM	BMMNCs	LVESV LVEDV, LVEDD, 6-MWT	Overall moderate risk of bias	Autologous bone marrow cell implantation significantly improves ventricular ejection fraction, end-diastolic diameter, and 6MWT outcomes.	No publication bias detected
Rong *et a*l.^37^	PubMed, Scopus, and Cochrane CENTRAL, November 2017	8	2010–2015	DCM	SCT	Mortality, LVEF, LVESV, LVEDCS, and 6-MWT.	Overall moderate risk of bias	Stem cell therapy enhances left ventricular ejection fraction and decreases both left ventricular end-systolic volume and chamber size in dilated cardiomyopathy patients. It doesn’t impact mortality or exercise capacity.	No publication bias detected for any outcome (begg’s test, egger test:- *P*>0.1)
Tripathi *et al*.^24^	Medline/Ovid, Embase, and Cochrane Central, August 2020	11	2010–2014	NIDCM	BMMNCs, BM-MSCs,	LVEF, LVEDD, NYHA classification, QoL, KCCQ, 6-MWT, and MACEs	Overall moderate risk of bias	In NICM patients on maximal medical therapy, cell therapy boosts LV systolic function and might reduce LV diastolic dimensions. it enhances functional capacity, evidenced by the 6-min walking distance. Cell therapy is deemed safe, with no rise in MACE compared to the control group	No publication bias detected
Wang *et al*.^38^	PubMed and EMBAS, March 2019	20	2006–2017	DCM	Bone marrow-derived cells	LVEF, LVEDV, LVESV, 6-MWT, NYHA class, QoL, MACE, all-cause mortality, and rehospitalization rate	Overall risk of bias was moderate to low		NA
Wen *et al*.^39^	PubMed, Scopus, and Cochrane CENTRAL	7	2008–2015	NIDCM	BM-MNCSs	LVEF, LVESV, All-cause mortality	Overall moderate risk of bias	Using BMMNCs is safe and may enhance LVEF and LVESV in non-ischaemic DCM but doesn’t reduce mortality compared to controls.	NA
Xia *et al*.^39^	PubMed, Scopus, and Cochrane CENTRAL, March 19, 2020	12	2006–2018	NIDCM	Analogues BM-MNCSs	Mortality and heart transplantation, LVEF, LVEDD, BNP and 6-WMT	Overall risk of bias was moderate to low	Cell therapy improved left ventricular contractility and exercise capacity without affecting chamber diameter. It decreased death and heart transplant rates, especially when delivered intracoronarily rather than intramyocardially.	For LVEF, LVEDD and 6-WMT funnel plot indicated asymmetry

6-MWT, 6-minute walk test; BM-MNCSs, bone marrow mononuclear cells; BM-MSCs, bone marrow mesenchymal stem cells; BNP, B-type natriuretic peptide; DCM, dilated cardiomyopathy; KCCQ, Kansas City Cardiomyopathy Questionnaire; LVEDCS, left ventricular end-diastolic chamber size; LVEDD, left ventricular end-diastolic diameter; LVEDV, left ventricular end-diastolic volume; LVEF, left ventricle ejection fraction; LVESV, Left Ventricular end-systolic volume; LVSV, left ventricular stroke volume; MACE, major adverse cardiovascular events; MLHFQ, Minnesota living with heart failure questionnaire; NIDCM, non-ischaemic dilated cardiomyopathy; NYHA, New York Heart Association; QoL, quality of life; SCT, stem cell therapy.

In the quality appraisal of the systematic reviews, only one study found to be of high quality. Three studies were seemed to be moderate quality and the remaining five were of low quality as per the JBI quality assessment tool (Table S5, Supplemental Digital Content 2, http://links.lww.com/JS9/B818).

### Summary of outcomes

Table 2 presents the summary of outcomes.

**Table 2:** Summary details of various outcomes.

Outcomes	Review name	No. studies	Total participants	Summary effect	Effect size (95% CI)	*p* value	Heterogeneity (I^2^)	Certainty of evidence
MACCE	Tripathi 2021	11	569	Odds ratio (MH, random, 95% CI)	0.77 [0.48, 1.24]	0.28	17%	Low
All-cause mortality	Xia 2020	12	623	Risk ratio (MH, fixed, 95% CI)	0.78 [0.55, 1.11]	0.17	0%	Very low
6-MWT (min)	Diaz-Navarro 2021	5	230	Mean difference (IV, random, 95% CI)	70.12 [−5.28, 145.51]	0.07	87%	Very low
HRQL	Diaz-Navarro 2021	5	272	Mean difference (IV, random, 95% CI)	0.62 [0.01, 1.23]	0.05	80%	Very low
LVEF (%)	Xia 2020	12	586	Mean difference (IV, random, 95% CI)	4.08 [1.93, 6.23]	0.0002	73%	Very low
LVEDD (cm)	Xia 2020	8	369	Mean difference (IV, random, 95% CI)	−0.09 [−0.23, 0.06]	0.25	54%	Very low
NYHA	Xia 2020	3	135	Mean difference (IV, random, 95% CI)	−0.70 [-0.98, −0.43]	<0.000001	29%	Very low
LVESV (ml)	Wang 2019	5	316	Mean difference (IV, random, 95% CI)	−21.29 [−33.20, −9.39]	0.0005	0%	Moderate
LVEDV (ml)	Wang 2019	6	466	Mean difference (IV, random, 95% CI)	2.35 [−6.42, 11.12]	0.60	18%	Low
BNP	Xia 2020	4	135	Mean difference (IV, random, 95% CI)	−326.66 [−749.24, 95.92]	0.13	92%	Very low

6-MWT, 6-minute walk test; BNP, B-type natriuretic peptide; HRQL, health-related quality of life; LVEDD, left ventricular end-diastolic diameter; LVEDV, left ventricular end-diastolic volume; LVEF, left ventricular ejection fraction; LVESV, left ventricular end-systolic volume; MACCE, major adverse cardiac and cerebrovascular events; NYHA, New York Heart Association Functional Classification.

### Left ventricular ejection fraction

The improvement in LVEF following stem cell therapy has been reported in numerous studies. Diaz-Navarro *et al*.^23^. investigated the effects of SCT on LVEF in participants with DCM and found no significant improvement compared to controls, with a mean difference (MD) of 5.41% (95% CI: −2.29 to 13.10; I^2^ = 94%; based on 8 studies with 353 participants). In contrast, Wen 2018 *et al*.^39^. observed that bone marrow mononuclear cells (BMMNCs) therapy significantly boosted LVEF by 3.79% (95% CI: 0.56–7.03%; *P* = 0.007). Wang *et al*.^38^. reported a marked improvement in LVEF by 3.72% (95% CI 2.31–5.13, *P* < 0.0001) in the bone marrow cell (BMC) group compared to controls. Tripathi *et al*.^24^. analyzed pooled data from nine trials and determined a 4.17% higher LVEF in the treatment group than in controls (95% CI = 1.66–6.69; *P* = .001; I^2^ = 48%). Both Rong *et al*.^37^. and Nso *et al*.^25^. reviewed the same data set of eight articles with 398 participants and emphasized the significant statistical heterogeneity in LVEF outcomes. Marquis-Gravel *et al*.^36^. illustrated a LVEF improvement of 4.87% points higher (95% CI: 1.32–8.43%) in the treatment group than in the control group (*P*=0.01). Lu *et al*.^26^. indicated a trend of LVEF improvement in the cell group at short-term follow-up, with an increase of 1.83% (95% CI –0.27 to 3.94, *P* = 0.09). However, during mid-term follow-up, bone marrow-derived SCT resulted in a significant LVEF enhancement of 3.53% (95% CI 0.76–6.29, *P* = 0.01). Furthermore, Xia *et al*.^40^. 2020 analyzed 12 RCTs involving 586 participants and discovered a mean difference of 4.08% in LVEF (95% CI: 1.93–6.32). The overall certainty of evidence was found to be very low (Table S6, Supplemental Digital Content 2, http://links.lww.com/JS9/B818).

### Left ventricular end-diastolic diameter

In the assessment of LVEDD, Marquis-Gravel *et al*.^36^. observed a treatment group decrease of -2.19 mm (95% CI: −5.69 to 1.30) with SCT. Meanwhile, Tripathi *et al*.^24^. noted 0.5 points benefit in the cell therapy group (SMD = 0.50; 95% CI: 0.95–0.06; *P* = 0.03). In contrast, Xia *et al*.^40^, using data from 369 participants across 8 RCTs, found a smaller mean difference in LVEDD of −0.09 (95% CI: −0.23 to 0.06, *P*=0.25) with a heterogeneity of I^2^ 54%. The overall certainty of evidence was found to be very low.

### Left ventricular end-diastolic volume

In evaluating the efficacy of specific therapies on LVEDV, both Wang and Wen *et al*.^38,39^. found no clear evidence of significant improvement over control treatments. Specifically, Wang observed that BMC therapy showed no superior effect in enhancing LVEDV at different follow-up intervals, with the most notable result being a slight increase of 2.35 (95% CI: −6.42 to 11.12) during the overall assessment period. Similarly, Wen *et al*.^39^. highlighted that BMMNCs therapy, despite its potential, did not yield evident improvements, with a marginal decrease characterized by a weighted mean difference of −20.60 ml (95% CI: −45.44 to 2.25). The overall certainty of evidence was found to be low.

### Left Ventricular end-systolic volume

In the realm of therapies focusing on, various studies present a consistent indication of potential advantages from stem cell-based treatments. Diaz-Navarro *et al*.^23^. combined findings from four studies with 251 participants, illustrating that stem cell therapy led to a significant Left Ventricular end-systolic volume (LVESV) reduction of −30.97 ml (95% CI: −54.18 to -7.75). Wang *et al*.^38^. analyzed 397 participants and identified a notable mean reduction of −21.29 (95% CI: -33.20 to −9.39). Their data further emphasized a substantial LVESV decrease during a 12–60-month follow-up due to BMC treatment, but not in the immediate 3-month duration. Lu *et al*.^26^. documented that BMC therapy resulted in a LVESV drop of −24.94 ml. Lastly, Rong *et al*.^37^, pooling data from five articles with 248 participants, identified a significant LVESV decrease (SMD= −0.36, 95% CI: −0.61 to −0.10) with SCT group when compared to controls. The overall certainty of evidence for Left Ventricular end-systolic volume was found to be moderate.

### B-type natriuretic peptide (BNP)

In assessing BNP concentrations following various interventions, several studies provide pertinent data. Xia *et al*.^40^. conducted a systematic review of four studies, which included 135 participants, and identified a pooled mean difference in BNP levels of −326.66 (95% CI: −749.24 to 95.92), with a heterogeneity of I^2^ at 92%, *P*=0.13. In another review, Tripathi *et al*.^24^. analyzed six trials, noting that three RCTs reported a significant reduction in NT pro-BNP levels within the SCT group, while two trials exhibited no discernible differences in either NT pro-BNP or BNP concentrations post-intervention. In Diaz-Navarro *et al*.^23^. review one trial observed a difference in mean BNP levels between SCT recipients and controls, with an MD (final means) of −124.20 ng/l (95% CI: −223.37 to −25.03). The certainity of evidece was found to be very low.

### Health-related quality of life (HRQL)

Several studies have explored the impact of cell treatment on HRQL in patients with heart-related issues. Tripathi *et al*.^24^ analyzed seven trials that employed various QoL. They found that two RCTs indicated an improvement in the QoL for the SCT group. However, when analyzing pooled data from four specific trials that used MLHFQ and KCCQ scores, no significant difference was observed between treatment and control groups, yielding a SMD of 0.13 (95% CI = 0.12–0.39; *P* = 0.30; I^2^ = 0%). In contrast, Wang *et al*.^38^, reported that QoL scores significantly dropped in the BMC treatment group compared to controls. Their pooled data from four trials showed a mean difference of −18.41 (95% CI: −29.90 to −6.92; *P* = 0.002). Diaz-Navarro *et al*.^23^, reviewing five studies involving 272 participants, concluded that the evidence remains uncertain about whether SCT enhances HRQL compared to controls. They presented an SMD of 0.62 (95% CI 0.01–1.23; I^2^ = 80%). The certainity of evidece was found to be very low.

### New York Heart Association

Tripathi *et al*.^24^ studied the effects of treatment on the NYHA classification across seven trials. They found that while two trials demonstrated a significant improvement in the NYHA classification for the treatment group, the remaining five trials suggested no significant correlation. On the other hand, Wang *et al*.^38^ observed that BMC treatment resulted in a noteworthy improvement in NYHA functional class, with a MD of −0.48 (95% CI: −0.65 to −0.31; *P* < 0.0001) compared to control group, spanning multiple follow-up durations. The overall certainty of evidence was found to be very low.

### 6-minute walk test

Tripathi *et al*.^24^ assessed the impact of treatment on the 6-minute walk distance in six trials. They found that two trials reported a significant enhancement in the SCT group over the control, while the remaining four indicated no meaningful change. However, a pooled analysis of data from five of these trials showcased a significant improvement in the 6-min walk distance post cell treatment, with a MD of 72.49 m (95% CI = 3.44–141.53; *P* = .04; I^2^ = 72%). In Wang *et al*.’s^38^ study, BMC treatment noticeably increased the 6-minute walk distance by 53.16 m (95% CI: 25.17–81.10) compared to controls (*P*=0.0002). This improvement was consistent across various follow-up durations ranging from 3 to 60 months. Diaz-Navarro *et al*.^23^ review of five studies, which included a total of 230 participants, explored the effect of SCT on exercise capacity measured by the 6-MWT. Their findings suggested a mean difference of 70.12 m (95% CI −5.28 to 145.51; I^2^ = 87%) when compared to controls. The overall certainty of evidence was found to be very low.

### Safety and mortality

In studies by Marquis-Gravel *et al*.^36^, there were no reported instances of periprocedural myocardial infarction or significant elevation of troponin levels. However, one of their trials did note two cases (10%) of periprocedural ventricular tachy-arrhythmias in the treatment group. Another trial highlighted two episodes (4%) of non-sustained ventricular tachycardia during intracoronary cell injection. In terms of mortality, a trial highlighted a 24% mortality rate in the treatment group versus a 30% rate in the control group over a mean follow-up of 28 ± 9 months^41^. One study observed 14% mortality in the treatment group and 35% in the control group after a 5-year follow-up. Meanwhile, another study reported a mortality rate of 35% in the SCT group and 10% in the control group after a 12-month follow-up^36^. The overall certainty of evidence for all-cause mortality was found to be very low.

The meta-analysis of pooled data by Tripathi *et al*.^24^ from different trials showed no significant difference in MACE event rates between the treatment and control groups. Yet, a sensitivity analysis, which excluded trials perceived to have a high risk of bias, pointed towards a decreased risk of MACE in the group undergoing cell therapy^24^.

Wang *et al*.^38^. reported all-cause death rates of 42% for the BMMNCs administration group and 17.1% for the control group. However, the difference in all-cause death risk between these groups wasn’t deemed significant. This study also touched upon MACE. While events such as cardiovascular disease related death, arrhythmia, sudden cardiac death, and myocardial infarction were distributed across the BMMNCs and control groups, most of the trials either didn’t mention procedure-related complications like arrhythmia and myocardial infarction or reported none^38^. The overall certainty of evidence for MACE is low.

## Discussion

This is the first umbrella review to summarize the safety and efficacy of SCT for DCM. Although many systematic reviews have explored this topic, none of the single reviews have comprehensively considered all outcomes. We summarized the evidence for all outcomes. Our synthesis showed that stem cell therapy may be effective for DCM. We found significant improvement in LVEF, NYHA, and LVESV. Other parameters also showed improvement, but the results were not significantly different overall. In terms of safety, SCT was generally found to be safe, without any MACE. Some studies showed a decrease in all-cause mortality rate, but it was not significant. The overall quality of evidence for most outcomes was found to be very low or low. The main limitation of the current evidence was the presence of a risk of bias in the RCTs.

Various systematic reviews have examined different outcomes, with a majority considering LVEF. Xia *et al*.^40^ determined that cell treatments through the intracoronary method could potentially enhance patient survival rates (combining death and heart transplantation instances), exercise capability (as measured by the 6-MWT), and heart ejection fraction (LVEF) for NICM. However, no significant change was observed in the cardiac chamber size (LVEDD). These findings hint at the potential of stem cell treatments as a viable alternative to conventional NICM therapies. Further extensive clinical studies, particularly well-designed RCTs, are crucial. For a comprehensive understanding of the impact of stem cell treatments on NICM, especially regarding mortality rates, we need more extensive data. Marquis-Gravel and team^36^ proposed that SCT could possibly elevate LVEF in non-ischaemic cardiomyopathy patients, but it might not have any significant effect on LVEDD.

There are various types of SCT. Mesenchymal stem cell (MSC) therapy has shown promise in regenerative therapy for acute and chronic illnesses, including ischaemic heart failure (IHF)^42^. However, the clinical benefit of MSC therapy is uncertain, and limited engraftment in the myocardium has been observed in long-term follow-up studies. The delivery routes for MSCs include trans-epicardial intramyocardial injection and cellular patches, which improve cell survival and engraftment. MSCs have been transplanted into the heart as autografts (from the patient’s own tissues) or allografts (tissue from another person). MSCs serve as a reservoir for cytokines and chemokines, which have advantageous paracrine effects in tissue healing. The underlying details of myocardial repair and the factors influencing the survival rate and efficacy of transplanted MSCs are still not fully understood. Similarly, the mechanism by which stem cell therapy might enhance heart function in DCM patients is not yet fully understood. Lin and colleagues^43^ found that cells in the LV myocardium, derived from stem cells, were not enough to support heart health, suggesting that the process behind stem cell therapy might be more intricate. In a separate study, Sun and his team^44^ treated DCM rats with stem cells from bone marrow and observed an improvement in LV function, primarily by reducing cell death. Many now think that the main way cell therapy works is through its indirect effects, releasing substances like cytokines, chemokines, and growth factors. These substances can prevent cell death, reduce scarring, improve muscle contraction, and stimulate the body’s own healing cells. However, more research is needed to pinpoint how cell therapy truly benefits DCM patients’ heart function.

The future of SCT for DCM holds immense promise and potential. As our understanding of the current evidence deepens, researchers are poised to explore novel approaches to harness the reparative capabilities of stem cells to reverse the myocardial damage and dysfunction associated with DCM^45^. Upcoming studies are likely to focus on refining the types of stem cells used, optimizing delivery methods, and determining the most effective dosages and treatment regimens. Additionally, the integration of advanced imaging techniques and biomolecular tools will enable a more precise assessment of stem cell engraftment, survival, and differentiation within the myocardium. Furthermore, combining stem cell therapy with other emerging treatments, such as gene therapy or precision medicine, may offer synergistic benefits. As clinical trials progress, there is hope that stem cell-based interventions will become a standard therapeutic option for DCM patients, reducing morbidity and mortality and improving the quality of life. However, more RCTs with stringent quality criteria should be conducted. Only then can concrete evidence be generated, and we can be certain about the benefits of SCT for DCM.

Although our review was able to summarize the available evidence on the topic, there are some limitations that should be acknowledged. We only included articles published in English. We could not perform a separate analysis by pooling data from primary studies because some of the trials included in these systematic reviews weren’t available in English. Secondly, there is some risk of bias in the included primary studies, which affected the certainty of the evidence. Future investigations should emphasize RCTs of high quality with larger sample sizes, more diverse patient populations, and longer follow-up periods. With the evolution of stem cell techniques and the identification of different stem cell sources, research should also focus on comparing the efficacy of various stem cell types and delivery methods.

## Conclusion

SCT for dilated cardiomyopathy has shown potential benefits in improving heart function, with several studies indicating its safety and efficacy. However, the current evidence is limited by biases in primary studies. Future research, emphasizing high-quality RCTS, is essential to validate the therapeutic benefits of SCT for DCM and to explore the most effective stem cell types and delivery methods.

## Ethical approval

Not applicable.

## Consent

Not applicable.

## Source of funding

This study received no funding.

## Author contribution

J.K.G. contributed to the study concept and design, and provided critical revisions to the paper. J.R. played a key role in data collection and also assisted with the study design. A.D. was involved in data analysis, interpretation, and provided substantial input in the writing of the manuscript. I.H.N. contributed to data collection and the writing of specific sections of the paper. R.I. focused on data analysis and contributed to the interpretation of the data. P.S. was involved in the study design, data interpretation, and provided oversight to the entire research process. M.N.K., S.R. contributed to data collection and played a role in drafting the initial manuscript. S.G., A.M.G. were instrumental in data analysis and also contributed to the critical revision of the manuscript for important intellectual content. Q.S.Z. assisted with data interpretation and was involved in revising the manuscript critically for significant intellectual content. R.S. oversaw the entire project, contributed to study conception, design, and also played a major role in writing and revising the manuscript.

## Conflicts of interest disclosure

The authors disclose no conflicts of interest

## Research registration unique identifying number (UIN)

PROSPERO: CRD42023473967 Research Registry (UIN: reviewregistry1781)

## Guarantor

Dr Ranjit Sah and Dr Prakasini Satapathy.

## Data statement

Data can be shared on request.

## Provenance and peer review

Not commissioned, externally peer-reviewed.

## Supplementary Material

SUPPLEMENTARY MATERIAL
